# Who spent their COVID-19 stimulus payment? Evidence from personal finance software in Japan

**DOI:** 10.1007/s42973-021-00080-0

**Published:** 2021-06-24

**Authors:** Michiru Kaneda, So Kubota, Satoshi Tanaka

**Affiliations:** 1 Money Forward, Inc., Tamachi Station Tower S 21F, 3-1-21 Shibaura, Minato, Tokyo 108-0023 Japan; 2 School of Political Science and Economics, Waseda University, 1-6-1 Nishiwaseda, Shinjuku-ku, Tokyo, 169-8050 Japan; 3 School of Economics, University of Queensland, Level 6 Colin Clark Building (39), Blair Dr, Saint Lucia, QLD 4072 Australia

**Keywords:** COVID-19, Unconditional cash transfer program, Marginal propensity to consume, Personal finance software data, Natural experiment, Japan, E21, G21, G51

## Abstract

In response to the COVID-19 crisis, governments worldwide have been formulating and implementing different strategies to mitigate its social and economic impacts. We study the household consumption responses to Japan’s COVID-19 unconditional cash transfer program. Owing to frequent delays in local governments’ administrative procedures, the timing of the payment to households varied unexpectedly. Using this natural experiment, we analyze households’ consumption responses to cash transfers using high-frequency data from personal finance management software that links detailed information on expenditure, income, and wealth. We construct three consumption measures: one captures the baseline marginal propensity to consume (MPC), and the other two are for the lower and the upper bound of MPC. Additionally, we explore heterogeneity in MPCs by household income, wealth, and population characteristics, as well as consumption categories. Our results show that households exhibit immediate and non-negligible positive responses in household expenditure. There is significant heterogeneity depending on various household characteristics, with liquidity constraint status being the most crucial factor, in line with the standard consumption theory. Additionally, this study provides policymakers with insights regarding targeted cash transfer programs, conditioning on labor income, and liquidity constraints.

## Introduction

In response to the Coronavirus disease 2019 (COVID-19) pandemic, many countries have conducted non-pharmaceutical interventions to restrict social and economic activities, such as enforcing social distancing, travel bans, school closures, patients’ quarantine, and large-scale lockdowns. These interventions have affected workers’ and families’ income and spending patterns, making it difficult for some of them to pay for essential goods. Consequently, there has been a growing demand for household income support to compensate for these losses. In addition, since the COVID-19 shock has disproportionately affected vulnerable workers, such as service sector employees, females, low-income families, and working mothers (Kikuchi et al. 2021), the primary role of fiscal stimulus packages has become social protection. To help people in need under these rapidly changing economic conditions, many countries have implemented policies such as expanding unemployment benefits and job retention programs. Some countries have also focused on immediate assistance: simplifying administrative procedures and providing cash transfers without strict conditions on economic and social characteristics such as income level and employment status.

The Japanese cash-transfer program, called the special cash payment (SCP), is the simplest among COVID-19 fiscal stimulus programs over the world. The government paid 100,000 Japanese yen (approximately 950 US dollars) to every individual, from babies to the elderly, living in Japan, regardless of their social and economic status. In most cases, the total amount for each family was deposited into the household head’s bank account. Owing to the historic magnitude of the economic downturn and the extraordinary budget size, an evaluation of this program is needed. Moreover, the policy design is ideal for obtaining economic evidence on households’ reactions to fiscal stimuli. In particular, filling this knowledge gap is important for policymakers, given that the COVID-19 crisis is far from over, and many countries, including Japan, might have to implement similar measures again in the near future. What is the overall effect on consumption? What kind of goods are purchased? Do they help vulnerable families purchase necessities, or do the rich buy luxury goods?

We study households’ responses to the SCP program using high-frequency transaction data from the Money Forward ME, a personal finance management service that allows users to keep track of banking accounts, asset holdings, credit card spending, cash payments, and digital point services. The dataset includes de-identified information about inflows and outflows from various financial accounts, detailed purchases by item, and individual heterogeneity in levels of income, assets, and population characteristics. We obtain the exact date of the SCP deposit and the subsequent household financial transactions and consumption patterns.

The crucial factor in estimating the pure consequence of the cash-transfer program is tackling it as a “natural experiment.” In Japan, bank account information is not connected to population records, such as individual identification numbers. To deposit the SCP, each local office needs to manually collect bank account numbers. This huge paperwork burden significantly delays the cash distribution process in some local offices. From the viewpoint of households, this lag leads to a nearly random timing of payment in the short term. We observe considerable heterogeneity in payment timings, ranging from May to August. This feature guarantees random assignment and removes possible statistical bias caused by macro-level consumption swings in response to COVID-19 infections.

To estimate the marginal propensity to consume (MPC) for SCP payments, we define the baseline measure of total consumption as well as its lower and upper bounds. We first define the lower bound of consumption as the sum of expenditures on food and necessities, services, non-durable and durable goods, payments, and other uncategorized spending explicitly recorded in Money Forward ME. Next, we construct the baseline measure of consumption as the lower bound plus the cash withdrawal net of the recorded cash payment. In 2019, according to the Ministry of Internal Affairs and Communications, 73.2% of consumer purchases were paid in cash. Although they were not recorded on the Money Forward ME system, some of them were manually input into the software by the users. Our baseline measure captures these purchases under the assumption that most of the new cash withdrawals responding to the SCP payment were used for consumption. Finally, we make the upper bound by summing up the baseline measure and other unclear transactions, possibly including both consumption and financial transfers.

Our results show an immediate increase in household consumption right after the SCP payment for all the baseline and upper/lower-bound cases. These consumption measures gradually declined after the initial spike. The implied MPCs are 0.16 for the baseline case, 0.06 for the lower bound, and 0.27 for the upper bound within 6 weeks of receipt. These numbers are lower than Baker et al. (2020), who study similar households’ transaction data to evaluate the U.S. COVID-19 cash transfer program, but higher than Japanese MPCs estimated from the past transfer programs (Shimizutani 2006; Hsieh et al. 2010). Moreover, we explore how household MPCs vary across categories of consumption. Most categories show significant increases in spending but these magnitudes are different.

We also examine MPC heterogeneity by income, asset holdings, and demographic characteristics. As the standard theory of intertemporal optimization of consumption indicates, liquidity constrained households significantly respond to the stimulus payment more. Since our dataset includes a rich set of both income and wealth information, it allows us to define liquidity-constrained households as those with less net liquid assets than their monthly labor income. The liquidity-constrained households clearly show a higher consumption response than the non-liquidity-constraint households in our data. The result is consistent with the recent literature on wealthy hand-to-mouth households (Kaplan et al. 2014).

A more practical policy-relevant result is about the heterogeneity of labor income. The actual 2020 SCP payment was uniform, but the government originally planned a targeting transfer to families whose income in 2020 was limited and had declined from 2019. We first examine the MPCs of the subsamples defined by the 2020 labor income quartile. We find a non-negligibly larger MPC of the lowest income groups than the others, which may justify the conditional cash transfer to economically disadvantaged families. Next, we conduct a counterfactual analysis of the original targeting plan. We identify needy households that would have received the targeting transfers under the income restrictions of the original policy. Contrary to looking at only 2020 labor income, the targeting policy does not find clear results of vulnerable families’ higher MPC. Since the original plan required each recipient’s labor income in 2020 to be below that in 2019, the target group would have eliminated people who would have had no labor incomes in both 2019 and 2020. This group would have had the largest MPC, while they would have been included in the non-targeted group.

There is a growing body of literature exploring the consumption responses to cash transfers under the COVID-19 crisis. For example, Baker et al. (2020) investigated consumption responses to the Coronavirus Aid, Relief, and Economic Security (CARES) Act cash transfer in the U.S. using personal finance management software data similar to ours. Their findings are consistent with our main results: households with low income and less liquid assets show higher MPCs. The CARES Act was also examined by Karger and Rajan (2020) using individual-level transaction data of debit cards. They found a large variance in MPCs among households. Those are partially captured by household characteristics, but a large fraction still remains unexplained. Misra et al. (2021) used the same data but ZIP code level. They find higher MPCs in areas of low-income and higher living costs. In contrast to these three papers using actual transaction records, Coibion et al. (2020) measured MPCs by a large-scale unique survey. The household responses depend on employment status and liquidity constraints. Outside of the U.S., Feldman and Heffetz (2021) investigated one-time and universal cash transfers in Israel. Their unique survey reveals that differences in income and financial conditions create small impacts on spending but significant effects on debt payments and donations. Bounie et al. (2020) also measured the MPCs in the COVID-19 crisis using French transaction data, although it was about a back-to-school allowance for parents.

This paper is also in the large body of the literature, which studies the response of household consumption to unanticipated changes in income using the quasi-experimental approach. The first such effort was made by Bodkin (1959) who examined the consumption behavior of war veterans after the receipt of an unexpected dividend from the National Service Life Insurance. More recently, Agarwal and Qian (2014) use the Singapore government’s Growth Dividend Program in 2011, which included one-time cash payout ranging from $40 to $700 per resident. Foreigners were excluded from this program and thus serve as the control group in the experiment. Parker et al. (2013) study the consumption responses to the economic stimulus payments of 2008. They exploit the random variations in the timing of receipt, which was determined by the final two digits of the recipient’s social security number (SSN), digits that are effectively randomly assigned.^1^ In Japan’s context, Shimizutani (2006) and Hsieh et al. (2010) are in this literature. They used the Japanese Government’s shopping coupon program and the 1998 tax cut, respectively.

The paper closest to ours is Kubota et al. (2020), which examined Japan’s SCP payment policy using a bank account data. To provide policy implications immediately, Kubota et al. (2020) considered only gross financial outflows from a bank account as an upper bound of consumption. Our study advances their result by looking at direct records of expenditure by category, as well as detailed asset and income information over various financial accounts. Our paper supports their main results that income conditions and liquidity constraints are significant factors in the MPC heterogeneity. Our paper is also related to Koga and Matsumura (2020) who study the heterogeneity of MPCs linking to financial status in Japan. They show the importance of liquidity asset holdings, homeownership, and mortgages on the MPC variation using both a quantitative macroeconomic model and empirical analyses. They estimated MPCs from annual household panel data by specifying the household’s income process and also obtained self-reported MPCs from survey data. Our approach significantly differs from their two empirical approaches, by exploiting a natural experiment and using high-frequency data. We nevertheless find similar results of liquidity asset holdings and homeownership. We complement their results by documenting the dynamics of consumption responses with our high-frequency data.

## Institutional background and model specification

### Japan’s special cash payment program

Japan’s first COVID-19 case was confirmed on January 16, 2020, and the infected person had returned from Wuhan, China. The number of COVID-19 cases grew slowly in Japan until the second half of February, after which it accelerated exponentially. The public worried that the pandemic was more severe than the observed data, given Japan’s weak surveillance and limited capacity for polymerase chain reaction (PCR) testing. As in many other countries, the Japanese government has implemented various measures to prevent the outbreak of COVID-19, including requesting nationwide school closure on February 27, and Japan’s first declaration of the state of emergency on April 7 for seven prefectures, including Tokyo.^2^ On April 16, the declaration was extended to the rest of the country for an indefinite period. It was a request-based lockdown with no penalties for social activities; however, this announcement effectively reduced the infection. The Japanese government eventually lifted the state of emergency for the whole country by May 25.

Although the COVID-19 cases were on average milder than in most other countries, Japan experienced a severe recession. The real gross domestic product (GDP) dropped by $10.3\%$ in the second quarter of 2020, which was mainly driven by a large decline in household consumption. The average hours of work fell by 3.9% and 9.3% in April and May, respectively. The economic crisis was exacerbated by the declaration of the state of emergency, which reduced people’s mobility by 20%, as evaluated by cell phone global positioning system (GPS) data (Watanabe and Yabu 2020, 2021).^3^ In particular, the public raised concerns about vulnerable workers in the face-to-face service sector (Kikuchi et al. 2021).

To mitigate the negative economic impact of COVID-19, Prime Minister Shinzo Abe approved a conditional cash transfer program on April 3, 2020, whose eligibility condition was determined by labor income in 2019 and 2020. However, on April 16, this plan was replaced by an unconditional transfer scheme due to a more practical suggestion by the coalition partner party, Komeito. This new unconditional transfer was the SCP program, which provided 100,000 Japanese yen (approximately 950 US dollars) to all residents in Japan without any condition on age, income, family size, or nationality. This amount is approximately 42% of the median monthly earned income of the Japanese full-time workers. Each municipality in Japan was responsible for distributing SCP payments. They determine the start date of application depending on their administrative capacity, by notifying all the households residing in the municipalities to apply for the SCP online or by mail. In the application, each household head was asked to provide a bank account number to receive the total payments for all the household members at once. After the evaluation, the total amount for all family members was deposited into the household head’s bank account.

The payment dates of the SCP varied across households due to the administrative capacity of local governments and the experience of office staff. Although the transfer started in the first week of May in some regions and most municipalities had distributed the application forms by the end of May, there were significant differences in the timing of payment across municipalities. According to a survey of 43 large municipalities, as of the third week of June, three cities had completed distribution to less than 10% of the residents, and eight had finished less than 20%. They cited the significant amount of time needed to reply to the numerous inquiries and to check mailed envelopes as reasons for the delay.^4^ However, six municipalities finished cash transfers to more than 80% of the residents in the same week. In addition, there was a significant difference with respect to the payment day, even if households submitted applications on the same day to the same local office. Since many households applied soon after the arrival of the submission form, a few hours difference in submission resulted in a lag of several days in payment. Furthermore, the submission methods caused variation in the timing of payments; for example, postal applications were significantly faster than online applications due to insufficient preparation.

**Fig. 1:**
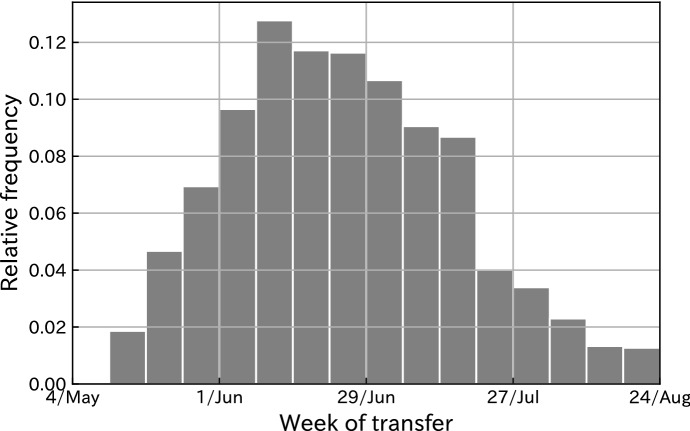
The distribution of the SCP payment week

Figure 1 shows the histogram of the number of SCP deposits to households in our dataset from May 4 and August 30. While the majority of the deposits occurred between late June and early July, the earliest payment started in May and the last payment in our sample was in the final week of August. As we discussed earlier, these variations in the timing of payments were largely driven by administrative delays, and can thus be regarded as unpredictable from the household’s perspective.

### Regression model specification

We exploit the variations in the timing of payments across households to estimate the effects of the SCP payments on their spending responses. Our regression has the following specifications:

1 $$\begin{aligned} y_{\mathrm{it}} = \alpha _i + \alpha _t + \sum\limits_{\begin{subarray}{l} k = k \\ k \ne \overline{{ - 1}} \end{subarray} }^{{\bar{k}}} {\gamma ^{k} P_{i} D_{{{\text{it}}}}^{k} } + \gamma ^{\mathrm{out}} P_{i} D^{\mathrm{out}}_{\mathrm{it}} + u_{\mathrm{it}}, \end{aligned}$$

where the dependent variable $y_{\mathrm{it}}$ is the spending by individual *i* in week *t*. Later, we introduce three measures of consumer spending to estimate the baseline and lower/upper MPCs. $\alpha _i$ represents individual fixed effects and control for time-invariant individual-specific factors. $\alpha _t$ are week fixed effects that control seasonally specific fluctuation of consumption, such as Christmas sales. $u_{\mathrm{it}}$ is the idiosyncratic error term.

$D_{\mathrm{it}}^k$ are indicators that take a value of one if the current week *t* is *k*-weeks after the week of the SCP transfer. $P_i$ is the amount of the SCP payment, which is a multiple of JPY 100,000 by the number of people in the household. We let $k \in \left[ {\underline{k}},{\overline{k}}\right]$ be the event time relative to the week of households’ SCP receipt. The previous week of the deposit corresponds to $k=-1$, and the week of payment is $k = 0$. Each coefficient $\gamma ^k$ (for $k >= 0$) captures the household’s dynamic spending responses to the fiscal payment at *k*-weeks after the deposit. We also examine the lead terms (for $k < -1$) to test for the presence of the pre-trend in the *k* weeks preceding the payment. In addition, we exclude $k=-1$ to normalize the coefficient of 1 week prior to SCP, $\gamma ^{-1}$, to 0. Thus, each $\gamma ^k$ for $k \ne -1$ can be interpreted as the consumption increase relative to $k=-1$. We also add one more dummy variable $D^{\mathrm{out}}_{\mathrm{it}}$ that controls effects outside the window; that is, $D^{\mathrm{out}}_{\mathrm{it}} = 1$ if the current week *t* is more than ${\underline{k}}$ weeks before the SCP transfer or more than ${\overline{k}}$ weeks after that. In our empirical analysis, we set ${\underline{k}}=-5$ and ${\overline{k}} = 10$.

Equation (1) is a standard specification for the econometric analysis of consumption responses to cash transfers. Similar specifications are used by Baker et al. (2020), Karger and Rajan (2020), and others.^5^

## Data

We describe the data for the estimation in this section.

### Money forward ME

We use de-identified transaction-level data from Money Forward ME, an online service, and a smartphone app for personal finance management. This service reports real-time transactions and visually represents monthly summaries of bank accounts, credit cards, securities, pensions, e-money, and retail shop points.^6^ Users can add up to ten financial accounts for free, or add unlimited accounts by paying JPY 500 (approximately USD 5) per month. All users can keep track of automatically recorded expenditures or manually input cash payments by consumption category.^7^

We first select users whose accounts recorded the SCP deposits. These accounts show income with the content name “special (TOKUBETU)” or “payment (KYUFU),” and the number multiples of JPY 100,000 between May 4 and August 30, 2020. Moreover, we choose only active users who have at least one transaction record every week between March 30 and November 8, 2020. We omit the top $1\%$ income users and top $1\%$ users of the maximum weekly payment. Finally, we get 232,589 users.

We convert the raw daily transaction data to account-level panel data of weekly balances and transactions. The dataset contains various asset holdings and account balances, such as demand-deposit accounts, saving accounts, mutual funds, bonds, including corporate, government bonds, and foreign, stocks, pensions, e-money, shop points, airline miles, forex, CFDs, cash, land, home, and precious metals. Money Forward ME also holds a rich dataset of debt information, including loans, such as car, mortgage, personal, and student, as well as credit card balances. In addition, we observe income information, such as labor income, business income, pension, stock dividends, and real-estate income. Finally, our dataset also contains some population characteristics, including sex, age, family structure, occupation, own/rent housing status, and residential prefecture.

### Asset, income, and population characteristics

Following the recent literature on household consumption, we construct gross/net and liquid/illiquid asset holdings (Kaplan et al. 2014). We first define gross liquid assets as cash, e-money, checking accounts, saving accounts, and securities. Net liquid assets are liquid assets minus credit card debt. We also define gross illiquid assets as the sum of real estate, cash value of life insurance, pension, and other uncategorized assets. Then, we calculate net illiquid assets as gross illiquid assets minus mortgage, student, and other loans. Finally, we define gross total assets as the sum of gross liquid and gross illiquid assets, and net total assets as the sum of net liquid and net illiquid assets.

We construct two measures to define household income. The first is wage and salary income, which are payments from employers explicitly recorded on the system or manually defined by users. This definition excludes the business income of the self-employed. The second is the total income, including financial and business income. We use the first measure as our benchmark in the main text and report the regression results with the second in “Appendix”. We estimate each user’s yearly income by doubling the sum of all incomes between April and September 2020.

In Table 1, we report the summary statistics of account holders’ assets, income, and population characteristics. First, the users are relatively young.^8^ Second, the share of female users is small, possibly because the SCP is paid to the household heads. Third, regarding location, many users live in Tokyo, as shown in Fig. 8 in “Appendix”.

Our income data are roughly consistent with public data around middle-income groups, but the variance is larger. The Family Income and Expenditure Survey (FIES) divides the sample of households into 0–20%, 20–40%, 40–60%, 60–80%, and 80–100% groups by their total household income, and provides the mean of the yearly after-tax income of each group. They are about 3.6, 4.7, 5.6, 6.8, and 9.2 million Japanese yen, respectively, for the sample of the households with at least two household members and with at least one employed member in 2020. In Money Forward Me, these figures are 2.4, 4.0, 5.3, 7.2, and 12.8. For the low-income groups, the bias could be because of the different structures of the two datasets. In particular, Money Forward Me might not be tracking the second earner’s income information well, which could create a downward bias in household income.^9^ On the other hand, our sample has a location bias to Tokyo. It could increase the average income of the high-income groups.

Our record of the wealth shows some differences from that of the FIES. This survey shows the mean gross liquid assets for 0–20%, 20–40%, 40–60%, 60–80%, and 80–100% groups of the sample of households with at two household members and with at least one employed member. They are 0.69, 2.65, 5.42, 10.37 and 30.47 million Japanese yen, respectively in 2020. In Money Forward ME’s data, they are 0.21, 1.14, 3.02, 7.27 and 32.12. The bottom column of Table 1 shows that approximately 40% of users answered “living in their own houses.” This number is comparable to the 61% measured in the Housing and Land Survey of Japan given that Money Forward ME users are biased to young.

**Table 1:** Summary statistics (account level)

	*N*	Mean	St. Dev.	25%	Median	75%
SCP payment (JPY)	232,589	225,294	131,464	100,000	200,000	300,000
Week of deposit	232,589	25.928	3.097	24	26	28
Age	228,644	42.967	91.813	31	37	45
Female dummy	229,810	0.282	0.450	0	0	1
Yearly labor income (JPY)	232,589	4,040,333	3,019,680	2,359,586	3,605,994	5,237,372
Yearly total income (JPY)	232,589	5,956,317	4,460,009	3,334,804	5,029,328	7,514,006
Gross liquid assets (JPY)	232,589	9,037,081	67,006,965	788,135	2,863,172	8,788,839
Net liquid assets (JPY)	232,589	8,638,142	67,008,374	483,642	2,555,419	8,454,560
Gross illiquid assets (JPY)	232,589	2,294,896	12,768,976	0	0	0
Net illiquid assets (JPY)	232,589	$-$ 2,983,116	15,783,263	0	0	0
Gross total assets (JPY)	232,589	11,331,977	69,128,440	843,462	3,189,144	10,229,096
Net total assets (JPY)	232,589	5,655,027	69,310,247	11,355	1,631,627	7,457,214
Own house dummy	147,046	0.398	0.489	0	0	1

### Expenditure

We classify household expenditures into six categories:

1. Food and necessities: Includes food made at home, daily necessities, and utilities.
2. Services: Our definition is slightly narrower than usual because we select services associated with possible new coronavirus transmission. It includes dining outside the home, transportation and travel, education, entertainment, and health care services. Given this definition, this category excludes home entertainment.
3. Non-durables: Includes non-durable goods, such as clothes, medicines, and home entertainment, except food and necessities and services.
4. Durables: Includes furniture, electric appliances, and cars.
5. Payments: Sum of loan, mortgage, rent, and insurance payments.
6. Uncategorized expenditures: Items that are not categorized as one of the above; however, they are bought at stores or paid for by credit cards, electronic payments, or cash.

Furthermore, we add two payment categories.

- ATM: This is the net amount of cash withdrawal from bank accounts mainly through ATMs. In Japan, cash is the dominant payment method. According to the Ministry of Internal Affairs and Communications, the share of cash payments was 73.2% in 2019. Moreover, the Japan Bankers Association reports that 49.1% of the outflow from bank accounts is cash withdrawal. Our definition is a partial net cash withdrawal because we deduct the amount of purchases by cash manually recorded by users from total cash withdrawals from their bank accounts. In other words, this ATM category includes two possibilities: cash payments not manually recorded and the amount of money saved in users’ wallets or strongboxes.
- Other transactions: This category includes taxes, social security payments, allowance for family members, business payments, and donations. In addition, there are other outflows from bank accounts.^10^ These outflows potentially include savings or investments if the bank account or the investment account is not registered at Money Forward ME.

Based on the above classification, we define the baseline measure and the lower and upper bounds of total consumption. First, we define the lower bound of consumption as the sum of all explicitly recorded expenditures.

Second, we define our baseline measure as the sum of the lower bound and ATM. Given that Japan is a cash economy, it is likely that the individual will eventually spend most cash withdrawals. Although we are unable to identify, we believe that the increase in the cash saving is limited, and most of them were actually used. According to the Survey of Household Finances conducted by the Central Council for Financial Services Information, the average cash savings of households with two or more members in 2020 is only about 0.2 million Japanese yen, compared to about 10 million Japanese yen in bank accounts. If households had kept the same portfolio after the SCP payments, the cash saving would have increased by only about 2000 Japanese yen. In addition, Fujiki and Tanaka (2018) and Fujiki et al. (2019) show that the cash holding is positively correlated with disposable income, but the coefficients are small. Under an assumption that the SCP is interpreted as an increase in disposable income, it raises cash savings about only 1000 Japanese yen.

Finally, we construct the upper bound of consumption including both baseline and other transactions. There are possibilities of underestimating true consumption expenditures under the baseline case, since some of those expenditures, such as bank transfers to stores, are potentially included in “other transactions.”

**Table 2:** Summary statistics (weekly transactions)

	*N*	Mean	St. Dev.	25%	Median	75%	FIES
Food and necessities	7,442,848	21,056	59,941	6380	14,375	26,798	24,651
Services	7,442,848	12,904	53,608	0	2473	11,128	18,989
Non-durable	7,442,848	9432	36,317	0	3575	10,016	10,001
Durable	7,442,848	6914	96,568	0	0	0	11,766
Payments	7,442,848	23,216	183,897	0	0	9568	22,001
Uncategorized expenditures	7,442,848	17,942	130,544	0	1100	10,000	–
ATM	7,442,848	14,459	84,340	0	0	0	($-$ 4479)
Other transactions	7,442,848	54,035	419,016	0	3131	21,685	28,499
Total expenditures	7,442,848	91,463	268,104	23,753	49,441	104,121	87,408
Total expenditures and ATM	7,442,848	105,922	284,483	27,514	57,424	119,918	–
All transactions	7,442,848	159,957	525,575	35,903	76,299	158,018	115,907

Notes: The last column reports the mean values of the 2020 FIES. The sample includes wage earners. We rearrange the small categories into our definitions to be consistent as much as possible. The ATM is calculated from cash holdings at the end of the survey month compared to the last month. It is excluded from calculating total values

Table 2 summarizes the weekly expenditure by consumption category. We also report the mean value calculated by the 2020 (FIES), in the last column. Overall, the mean expenditures of Money Forward ME users are consistent with the result of the public survey. However, our values are somewhat higher, possibly because Money Forward ME has a relatively smaller number of old-age users who tend to consume less. Another potential reason is the response bias, since the FIES requires respondents to fill in all payments to the survey sheets by hand, which may cause respondents to record fewer amount of purchases, compared to Money Forward ME’s automatic recording system. We record 91,463 Japanese yen on average as the amount of weekly consumption. After adding other transactions, the total weekly transaction is 159,957 Japanese yen. The purchase is not so frequent because the first quartile is zero, except for food and necessities. The frequencies of durable good purchases and cash withdrawals are once or less a month.

## Results

In this section, we present the estimation results of regression (1).

### Benchmark results

**Fig. 2:**
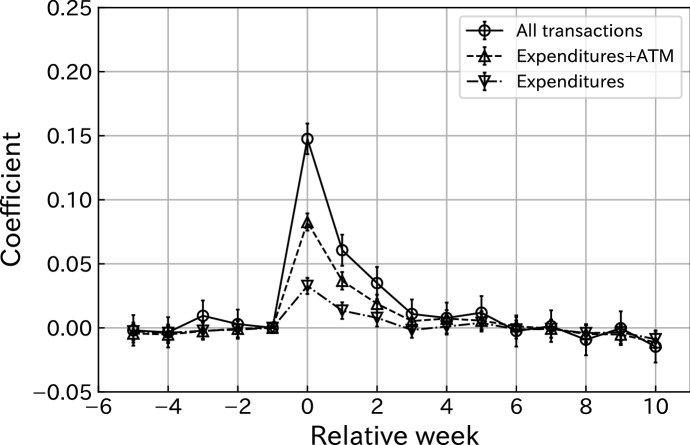
Consumption responses of all users. Notes: This figure plots the estimated coefficients ${\hat{\gamma }}^k$ for $k \in \{-5,\ldots ,-1, 0, 1, \ldots , 10\}$, where $\gamma ^{-1}$ is restricted to 0 in Eq. (1). Bars indicate 95% confidence intervals. Standard errors are clustered at the user level. The sample size is 232589

Figure 2 illustrates the estimates of $\gamma _k$ for the three regressions with different dependent variables. In figure, Expenditures+ATM draws our baseline consumption measure’s response to the SCP receipts. Similarly, the responses of the lower and upper bounds of consumptions are represented by Expenditures and All transactions, respectively.

Figure 2 shows a clear spike in consumption response right after the receipt of the payment, evaluated by all three measures.^11^ The positive effect gradually declines and persists for roughly 6 weeks. These weekly estimates for the coefficients are shown in Table 3. The estimated cumulative MPCs for 6 weeks are 0.06, 0.16, and 0.27 for the lower measure, the benchmark, and the upper measure, respectively.

**Table 3:** Regression results of all users

Relative week	All transactions	Expenditures+ATM	Expenditures
$-$ 5	$-$ 0.0020	$-$ 0.0044	$-$ 0.0028
	(0.0061)	(0.0036)	(0.0035)
$-$ 4	$-$ 0.0035	$-$ 0.0052	$-$ 0.0039
	(0.0060)	(0.0034)	(0.0033)
$-$ 3	0.0094	$-$ 0.0026	$-$ 0.0023
	(0.0061)	(0.0034)	(0.0032)
$-$ 2	0.0029	$-$ 0.0007	$-$ 0.0014
	(0.0058)	(0.0034)	(0.0033)
$-$ 1	0.0000	0.0000	0.0000
	(0.0000)	(0.0000)	(0.0000)
0	0.1475	0.0826	0.0327
	(0.0061)	(0.0034)	(0.0032)
1	0.0606	0.0367	0.0134
	(0.0062)	(0.0035)	(0.0033)
2	0.0349	0.0188	0.0078
	(0.0064)	(0.0036)	(0.0035)
3	0.0108	0.0053	$-$ 0.0017
	(0.0058)	(0.0032)	(0.0031)
4	0.0077	0.0072	0.0010
	(0.0061)	(0.0034)	(0.0032)
5	0.0118	0.0056	0.0039
	(0.0066)	(0.0037)	(0.0035)
6	$-$ 0.0025	0.0012	$-$ 0.0013
	(0.0062)	(0.0037)	(0.0036)
7	0.0013	$-$ 0.0006	$-$ 0.0009
	(0.0063)	(0.0036)	(0.0035)
8	$-$ 0.0093	$-$ 0.0049	$-$ 0.0041
	(0.0062)	(0.0035)	(0.0034)
9	$-$ 0.0003	$-$ 0.0052	$-$ 0.0035
	(0.0067)	(0.0036)	(0.0034)
10	$-$ 0.0147	$-$ 0.0114	$-$ 0.0089
	(0.0063)	(0.0036)	(0.0035)
Outside	$-$ 0.0156	$-$ 0.0117	$-$ 0.0079
	(0.0048)	(0.0028)	(0.0027)
Observations	7442848	7442848	7442848
$R^2$	0.0002	0.0003	0.00005

Notes: This table reports coefficients from Eq. (1). Standard errors are reported in parentheses and clustered at the user level. The coefficient of 1 week prior to the SCP is restricted to 0 in Eq. (1)

### Heterogeneous response by individual characteristics

Next, we turn to heterogeneity in the consumption responses among individuals based on their observable characteristics. The literature on MPC documents significant heterogeneity among households in their consumption response to transitory income shocks (Misra and Surico 2014; Alan et al. 2018; Parker 2017; Aguiar et al. 2020; Gelman 2021). Studies have reported heterogeneous consumption responses across recipients in the context of stimulus packages for COVID-19 (Baker et al. 2020; Coibion et al. 2020; Karger and Rajan 2020; Misra et al. 2021; Chetty et al. 2020). Therefore, we explore the heterogeneity in consumption response with respect to households’ labor income, financial constraints, and other observable characteristics.

**Fig. 3:**
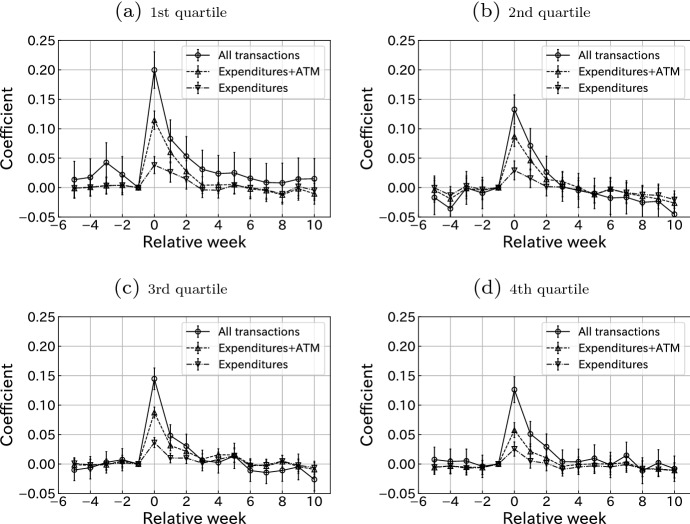
Consumption responses grouped by labor income. Notes: This figure plots the estimated coefficients ${\hat{\gamma }}^k$ for $k \in \{-5,\ldots ,-1, 0, 1, \ldots , 10\}$, where $\gamma ^{-1}$ is restricted to 0 in Eq. (1). Bars indicate 95% confidence intervals. Standard errors are clustered at the user level. The sample size of each group is 58147

Figure 3 shows the consumption response for each quartile group by labor income. The bottom quartile group shows the strongest consumption response, whereas the other three quartile groups show similar responses, implying that heterogeneity is more relevant for those in the low-income group rather than in the middle or high ones.

This heterogeneity may be larger. As we discussed in Sect. 3.2, low-income groups may include middle-income households whose second earner’s income are missing. This bias possibly lowers the MPC of Panel (a) in Fig. 3.

**Fig. 4:**
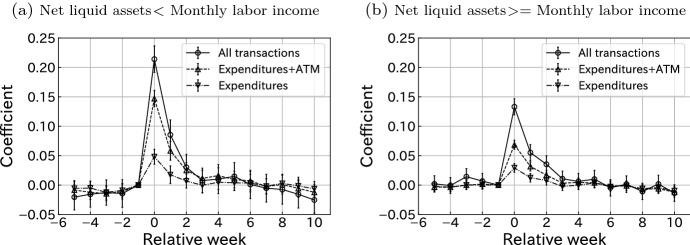
Consumption responses grouped by liquidity constraints using labor income. Notes: This figure plots the estimated coefficients ${\hat{\gamma }}^k$ for $k \in \{-5,\ldots ,-1, 0, 1, \ldots , 10\}$, where $\gamma ^{-1}$ is restricted to 0 in Eq. (1). Bars indicate 95% confidence intervals. Standard errors are clustered at the user level. The sample size of **a** is 44433 and that of **b** is 188156

We consider the liquidity constraint in Fig. 4. This is a crucial factor in determining MPC. If a household has insufficient assets and difficulty borrowing money, it may use a large portion of cash transfers to smooth intertemporal consumption allocation. We classify a user to be liquidity-constrained if his/her net liquid asset holding is less than his/her monthly labor income at the end of the month before the SCP receipt.^12^ In our dataset, 19% of users were liquidity constrained under this definition. Figure 4 shows consumption responses with respect to the individual liquidity constraint status. By comparing the left and right panels of the figure, it is clear that liquidity-constrained households respond more sharply to SCP payments, especially in the transfer week. The results confirm the important role of liquidity constraints in illustrating heterogeneity in consumption responses, as documented in the literature.^13^ For example, our result is consistent with Kubota et al. (2020), who study the same SCP program with different definitions of consumption and dataset. Figure 9 in “Appendix” plots the consumption response by liquidity constraint status and housing status (owning or renting). It shows that households with a liquidity constraint respond more than households without a constraint, even when they own a house. This result confirms the existence of wealthy hand-to-mouth households documented in the literature (Kaplan et al. 2014).

In addition, we explore heterogeneity by households’ other observable characteristics. The results are reported in “Appendix”. Figures 10, 11, and 12 show the consumption responses by age, family size, and family type, respectively. We find that the consumption response is larger if the household head is older and that one-person households’ responses are weaker than those of other households. We also find that married households respond more than single households, while having a child in a household does not seem to affect the consumption response.

### Heterogeneous responses across consumption categories

**Fig. 5:**
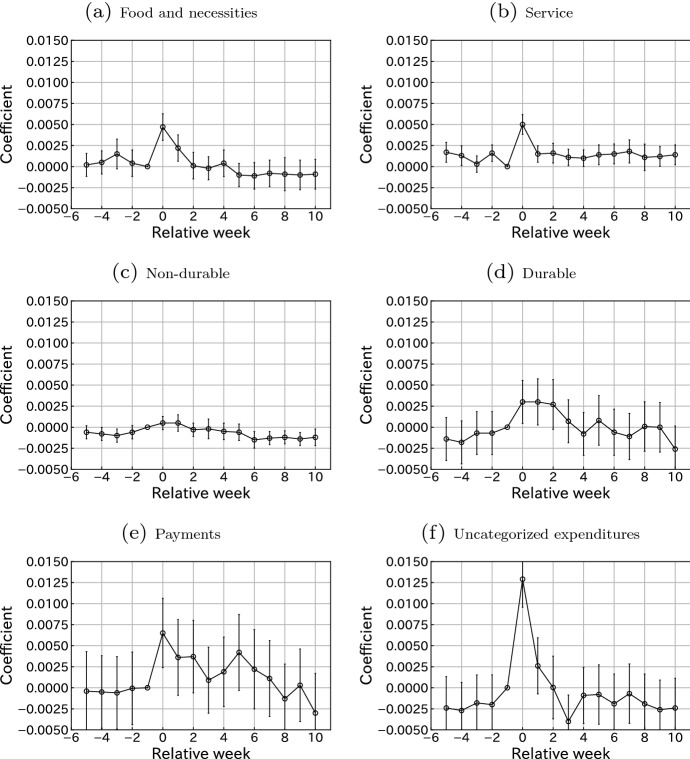
Consumption responses for each expenditure category. Notes: This figure plots the estimated coefficients ${\hat{\gamma }}^k$ for $k \in \{-5,\ldots ,-1, 0, 1, \ldots , 10\}$, where $\gamma ^{-1}$ is restricted to 0 in Eq. (1). Bars indicate 95% confidence intervals. Standard errors are clustered at the user level. The sample size is 232589

Next, we examine the responses by consumption categories. Figure 5 shows that payments and uncategorized expenditures exhibit large responses. These consumptions are volatile, as suggested by the large standard deviations in Table 2. The SCP may stimulate households to purchase special, occasional, and expensive items. The response of durable goods looks moderate but is actually large, given that its average monthly spending is low (6914 Japanese yen). Our results indicate an approximately $15\%$ increase in monthly durable good spending, which is lower but comparable to the 27.4% and 16.6% increase in the FIES in June and July 2020, respectively.^14^ We also find a statistically significant rise in the consumption of food and necessities, and services. Contrary to durable goods, the fluctuations of these items are unclear in the FIES. This is an advantage of analysis with a valid identification strategy using natural experiments and detailed microdata. The increase in spending on service is also notable under suppressed service demand due to the COVID-19 pandemic. Finally, we do not find an increase in the consumption of non-durable goods.

### Counterfactual policy analysis

**Fig. 6:**
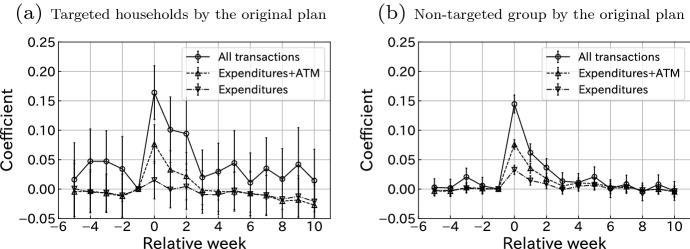
Counterfactual policy analysis: comparing consumption responses for targeted and non-targeted households according to the initial plan using labor income. Notes: This figure plots the estimated coefficients ${\hat{\gamma }}^k$ for $k \in \{-5,\ldots ,-1, 0, 1, \ldots , 10\}$, where $\gamma ^{-1}$ is restricted to 0 in Eq. (1). Bars indicate 95% confidence intervals. Standard errors are clustered at the user level. The sample size of **a** is 12967 and that of **b** is 142565

In response to the COVID-19 crisis, the Japanese government initially planned a targeted cash transfer program. On April 3, 2020, Prime Minister Shinzo Abe announced an emergency economic stimulus package of 300,000 Japanese yen for every household whose income had declined significantly due to the COVID-19 crisis. Specifically, Mr. Abe planned to target households (i) whose income from February through June 2020 had decreased compared to the same periods in 2019, and whose monthly income is equal to or less than the residential tax exemption cutoff, or (ii) whose income from February through June 2020 had dropped to lower than half of their 2019 income, and whose monthly income is equal to or less than the double of the residential tax exemption cutoff.^15^ We conduct our counterfactual analysis by estimating the MPC for the households targeted by the initial plan. Although Mr. Abe’s plan was replaced by the universal cash transfer program with a lower payment amount, it is important for policymakers and researchers to explore a counterfactual scenario with the original, targeted transfer program. This exercise also has implications for the ongoing debate regarding the second-round stimulus payment program. As of March 2021, as we are writing this paper, the next cash transfer program is still one of the most debated policy issues. On February 9, 2021, 79 Diet members of the Liberal Democratic Party requested conditional cash transfers for economically distressed families. Our counterfactual exercise can provide policymakers with insights regarding this issue.

Figure 6 plots the results of the estimations. In terms of all transactions, the consumption response by the targeted group shows a higher spike compared to that of the non-targeted group, especially 1 or 2 weeks after the SCP receipt. However, it is unclear because All transactions of the targeted group show significantly positive coefficients before the SCP deposit and the large standard errors. This result may be caused by a sample selection problem that the targeted group also includes high-income households whose salaries and financial accounts are not correctly recorded in our database. In terms of Expenditure and Expenditure+ATM, we do not see a clear difference between the two estimates. Figure 6 looks inconsistent with the previous result of heterogeneity based on labor income level in Fig. 3. The reason for the difference is that, under Mr. Abe’s initial plan, there is a restriction that 2020 household labor income must be below that in 2019. This restriction eliminates people who have no labor incomes both in 2019 and 2020. This group has the largest MPC, while they are included in the non-targeted group. Therefore, even if a policy targets households who were supposed to be covered by the initial plan, the average consumption response per recipient would not be higher than that of the universal program implemented by the Japanese government. To summarize, the policy consequences may be very sensitive to any small change in details. A simpler policy, such as the contingent on only the current labor income, may be more predictable and intuitive, as shown in Fig. 3.

## Conclusion

This study examines the effects of the Japanese unconditional cash transfer program on consumption using high-frequency information on assets, income, and expenditure obtained from personal financial management software data, provided by Money Forward ME. Owing to the significant delay in local governments’ administrative procedures, there has been a significant and unexpected variation in the timing of payment. Using this natural experiment, we estimate the pure effects of the stimulus payment package on household consumption.

Our results demonstrate significant heterogeneity depending on various household characteristics and highlight liquidity constraints as the most crucial factor, which is consistent with the standard consumption theory. These findings indicate the potential effectiveness of targeting policies depending on liquidity constraints; however, it might be unrealistic for the Japanese government to identify household wealth information. Moreover, we find that labor income inequality has a large impact on households’ consumption responses. In addition, we examine household responses across consumption categories. Most categories show significant increases in spending but these magnitudes are different. Finally, we analyze the Japanese government’s original targeting policy contingent on labor income as a counterfactual exercise, and find that the policy effects exhibit high sensitivity to policy details. Our results would be useful in future policy discussions.

## Figures and Tables

**Fig. 7:**
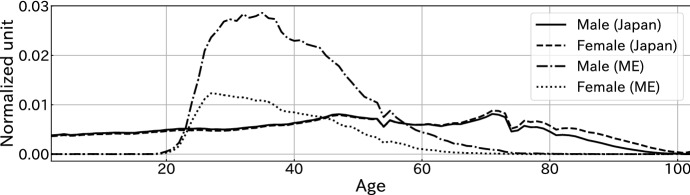
Age composition of population. Notes: The solid and dashed lines show the male and female populations in Japan, respectively, taken from the census data. The dot-dashed and dotted lines show the male and female population of the Money Forward ME users, respectively, used in this analysis. Both Japanese and Money Forward ME user data were normalized so that the total number of males and females is 1

**Fig. 8:**
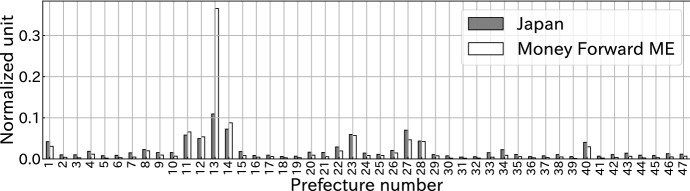
Normalized population by prefecture. Notes: The filled and unfilled bars represent the population of each prefecture in Japan taken from the census data and the Money Forward ME users used in this analysis. Both data were normalized so that the total number is 1

**Fig. 9:**
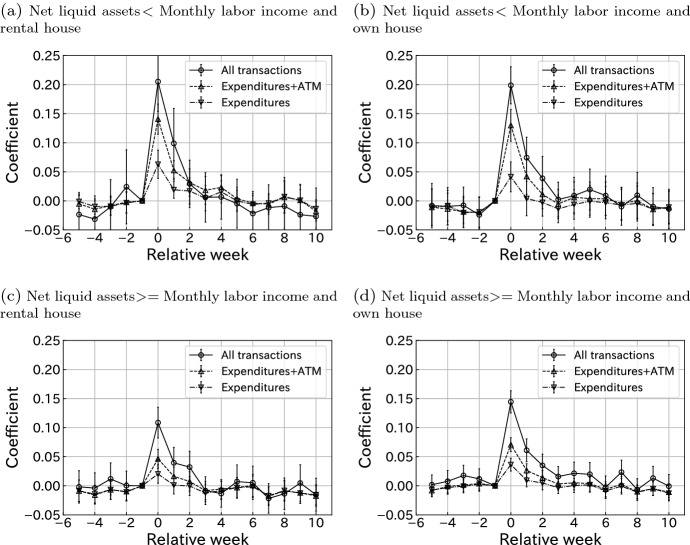
Consumption responses grouped by liquidity constraints using labor income and housing status. Notes: This figure plots the estimated coefficients ${\hat{\gamma }}^k$ for $k \in \{-5,\ldots ,-1, 0, 1, \ldots , 10\}$, where $\gamma ^{-1}$ is restricted to 0 in Eq. (1). Bars indicate 95% confidence intervals. Standard errors are clustered at the user level. The sample size of **a**–**d** are 18046, 9026, 70544, and 49430, respectively

**Fig. 10:**
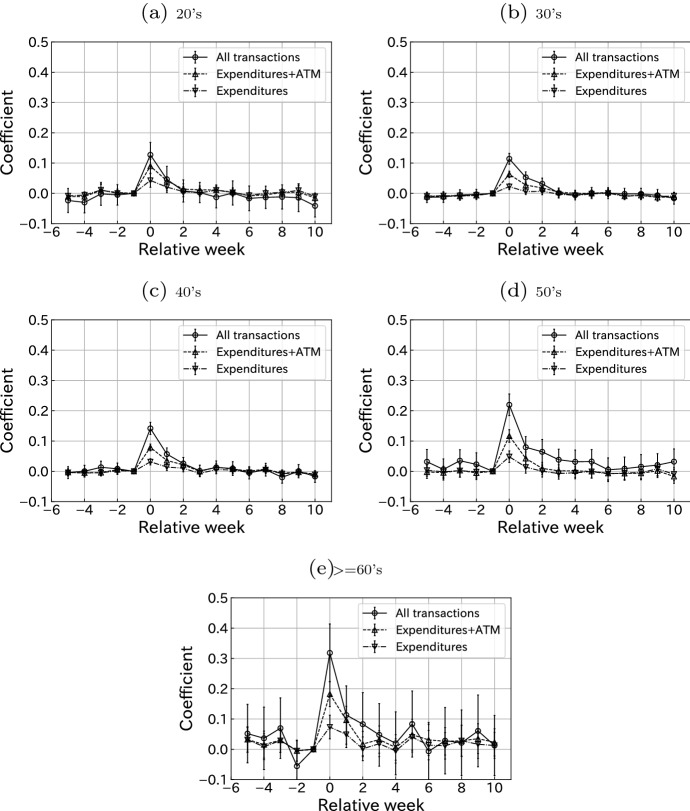
Consumption responses grouped by age. Notes: This figure plots the estimated coefficients ${\hat{\gamma }}^k$ for $k \in \{-5,\ldots ,-1, 0, 1, \ldots , 10\}$, where $\gamma ^{-1}$ is restricted to 0 in Eq. (1). Bars indicate 95% confidence intervals. Standard errors are clustered at the user level. The sample size of **a**–**e** are 45955, 85733, 61710, 26214, and 8928, respectively

**Fig. 11:**
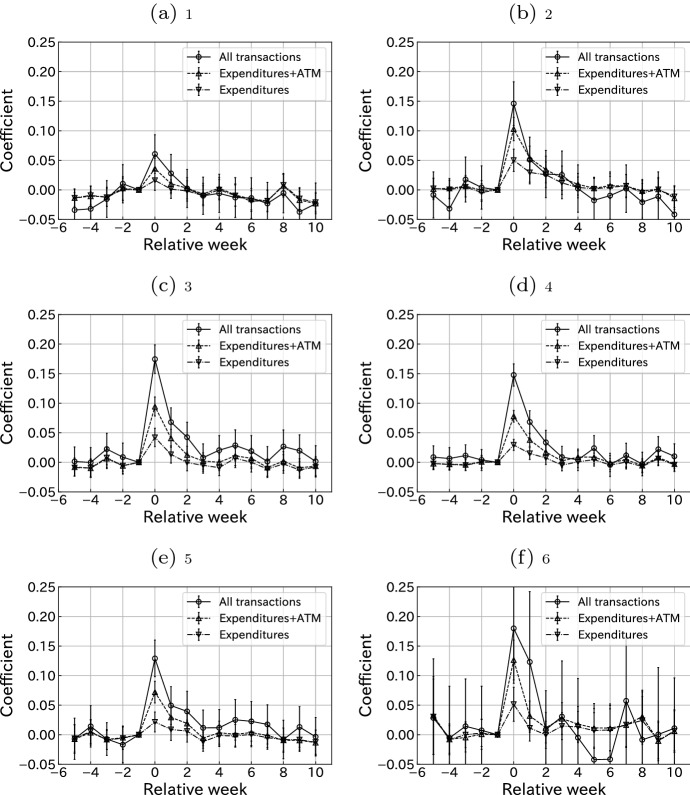
Consumption responses grouped by family size. Notes: This figure plots the estimated coefficients ${\hat{\gamma }}^k$ for $k \in \{-5,\ldots ,-1, 0, 1, \ldots , 10\}$, where $\gamma ^{-1}$ is restricted to 0 in Eq. (1). Bars indicate 95% confidence intervals. Standard errors are clustered at the user level. The sample size of **a**–**f** are 98996, 40704, 40895, 40812, 9731, and 1197, respectively

**Fig. 12:**
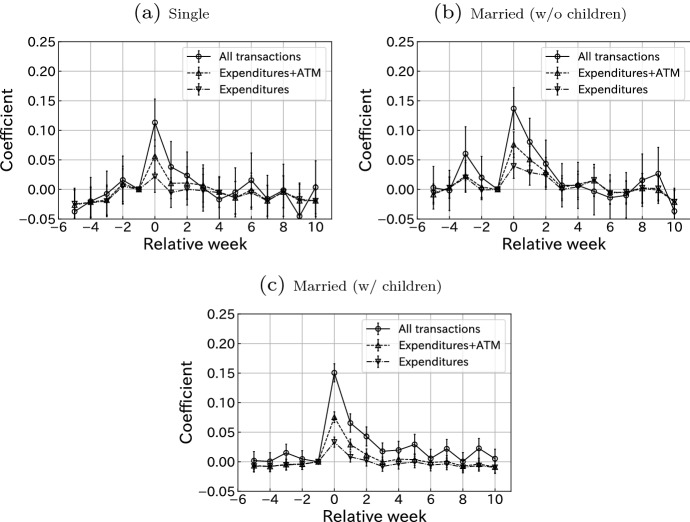
Consumption responses grouped by family type. Notes: This figure plots the estimated coefficients ${\hat{\gamma }}^k$ for $k \in \{-5,\ldots ,-1, 0, 1, \ldots , 10\}$, where $\gamma ^{-1}$ is restricted to 0 in Eq. (1). Bars indicate 95% confidence intervals. Standard errors are clustered at the user level. The sample size of **a**–**c** are 66154, 28197, and 68042, respectively

**Fig. 13:**
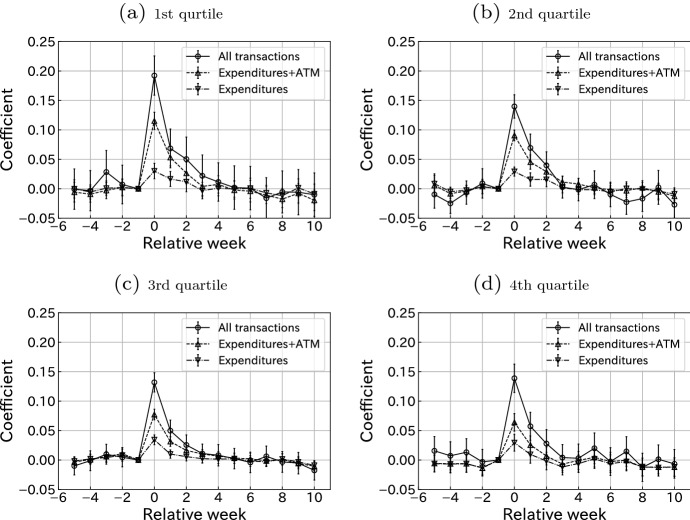
Consumption responses grouped by total income. Notes: This figure plots the estimated coefficients ${\hat{\gamma }}^k$ for $k \in \{-5,\ldots ,-1, 0, 1, \ldots , 10\}$, where $\gamma ^{-1}$ is restricted to 0 in Eq. (1). Bars indicate 95% confidence intervals. Standard errors are clustered at the user level. The sample size of each group is 58147

**Fig. 14:**
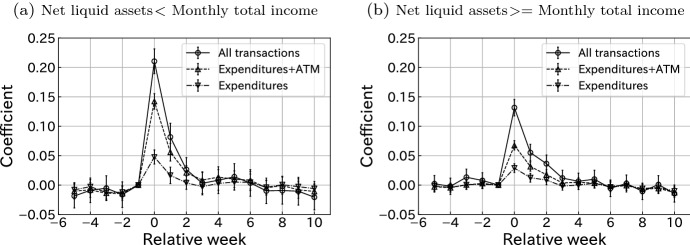
Consumption responses grouped by liquidity constraints using total income.

**Fig. 15:**
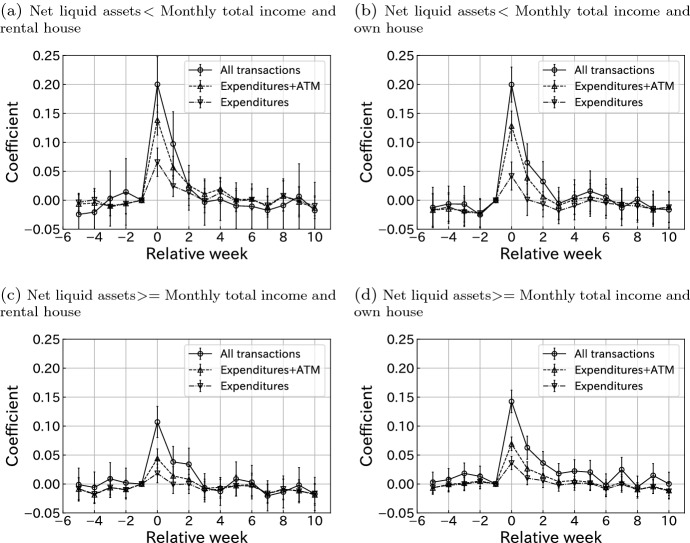
Consumption responses grouped by liquidity constraints using total income and house status. Notes: This figure plots the estimated coefficients ${\hat{\gamma }}^k$ for $k \in \{-5,\ldots ,-1, 0, 1, \ldots , 10\}$, where $\gamma ^{-1}$ is restricted to 0 in Eq. (1). Bars indicate 95% confidence intervals. Standard errors are clustered at the user level. The sample size of **a**–**d** are 20191, 10314, 68399, and 48142, respectively

**Fig. 16:**
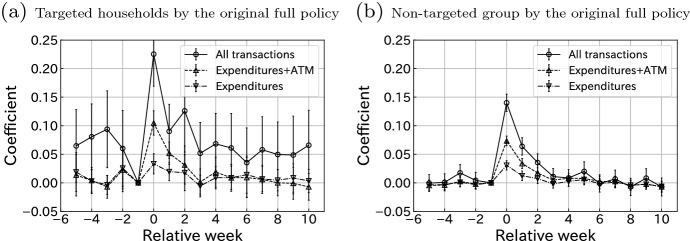
Counterfactual policy analysis: comparing consumption responses for targeted and non-targeted households according to the initial plan using total income. Notes: This figure plots the estimated coefficients ${\hat{\gamma }}^k$ for $k \in \{-5,\ldots ,-1, 0, 1, \ldots , 10\}$, where $\gamma ^{-1}$ is restricted to 0 in Eq. (1). Bars indicate 95% confidence intervals. Standard errors are clustered at the user level. The sample size of **a** is 10871 and that of **b** is 144661

## Appendix

### Population distribution

See Figs. 7, 8.

### Additional figures and tables

See Figs. 9, 10, 11, 12 and Table 4.

**Table 4:** Regression results for each consumption category

Relative week	Food and necessities	Services	Non-durable	Durable	Payments	Uncategorized expenditures
$-$ 5	0.0002	0.0017	$-$ 0.0006	$-$ 0.0014	$-$ 0.0004	$-$ 0.0024
	(0.0007)	(0.0006)	(0.0004)	(0.0013)	(0.0024)	(0.0019)
$-$ 4	0.0005	0.0013	$-$ 0.0008	$-$ 0.0018	$-$ 0.0005	$-$ 0.0027
	(0.0007)	(0.0006)	(0.0004)	(0.0013)	(0.0022)	(0.0017)
$-$ 3	0.0015	0.0003	$-$ 0.0010	$-$ 0.0007	$-$ 0.0006	$-$ 0.0018
	(0.0009)	(0.0005)	(0.0004)	(0.0013)	(0.0022)	(0.0017)
$-$ 2	0.0004	0.0016	$-$ 0.0006	$-$ 0.0007	$-$ 0.0001	$-$ 0.0020
	(0.0008)	(0.0005)	(0.0004)	(0.0013)	(0.0022)	(0.0018)
0	0.0047	0.0050	0.0005	0.0030	0.0065	0.0129
	(0.0008)	(0.0006)	(0.0004)	(0.0013)	(0.0021)	(0.0017)
1	0.0022	0.0015	0.0005	0.0030	0.0036	0.0026
	(0.0008)	(0.0005)	(0.0005)	(0.0014)	(0.0023)	(0.0017)
2	0.0001	0.0016	$-$ 0.0003	0.0027	0.0037	0.0000
	(0.0008)	(0.0006)	(0.0004)	(0.0015)	(0.0022)	(0.0019)
3	$-$ 0.0002	0.0011	$-$ 0.0002	0.0007	0.0009	$-$ 0.0040
	(0.0007)	(0.0005)	(0.0006)	(0.0013)	(0.0020)	(0.0016)
4	0.0004	0.0010	$-$ 0.0005	$-$ 0.0008	0.0019	$-$ 0.0009
	(0.0008)	(0.0005)	(0.0005)	(0.0013)	(0.0021)	(0.0017)
5	$-$ 0.0010	0.0014	$-$ 0.0006	0.0008	0.0042	$-$ 0.0008
	(0.0007)	(0.0006)	(0.0005)	(0.0015)	(0.0023)	(0.0018)
6	$-$ 0.0011	0.0015	$-$ 0.0015	$-$ 0.0006	0.0022	$-$ 0.0019
	(0.0008)	(0.0006)	(0.0005)	(0.0014)	(0.0024)	(0.0018)
7	$-$ 0.0008	0.0018	$-$ 0.0013	$-$ 0.0011	0.0011	$-$ 0.0007
	(0.0008)	(0.0007)	(0.0004)	(0.0014)	(0.0023)	(0.0018)
8	$-$ 0.0009	0.0011	$-$ 0.0012	0.0001	$-$ 0.0013	$-$ 0.0019
	(0.0010)	(0.0008)	(0.0004)	(0.0015)	(0.0021)	(0.0018)
9	$-$ 0.0010	0.0012	$-$ 0.0014	$-$ 0.0000	0.0003	$-$ 0.0026
	(0.0009)	(0.0006)	(0.0004)	(0.0015)	(0.0022)	(0.0018)
10	$-$ 0.0009	0.0014	$-$ 0.0012	$-$ 0.0026	$-$ 0.0030	$-$ 0.0024
	(0.0009)	(0.0006)	(0.0005)	(0.0014)	(0.0024)	(0.0018)
Outside	$-$ 0.0007	0.0023	$-$ 0.0015	$-$ 0.0023	$-$ 0.0019	$-$ 0.0036
	(0.0007)	(0.0005)	(0.0004)	(0.0012)	(0.0017)	(0.0015)
Observations	7442848	7442848	7442848	7442848	7442848	7442848
$R^2$	0.00002	0.00002	0.00002	0.00001	0.0000070	0.00003

Notes: This table reports coefficients from Eq. (1). Standard errors are reported in parentheses and clustered at the user level. The coefficient of 1 week prior to the SCP is restricted to 0 in Eq. (1)

### Results using total income

See Figs. 13, 14, 15, 16.

## Footnotes

1 Jappelli and Pistaferri (2017) provide a concrete survey of this literature.

2 The other prefectures for which the Japanese government declared the state of emergency are Kanagawa, Saitama, Chiba, Osaka, Hyogo, and Fukuoka.

3 These studies found the information effect to have the largest impact on the public, as the state of emergency revealed the severity of the pandemic.

4 Asahi Shinbun, morning edition, June 28.

5 The dummy for restricting effects window, $D^{\mathrm{out}}_{\mathrm{it}}$, is assumed the same before and after that in our specification. We select it for simplification. The results are almost unchanged if we allow the different coefficients before/after the effects window.

6 Credit card transactions potentially have a measurement issue in payment timing. If a user uses a credit card that is not linked to the app, a credit card payment shows up in the bank account when the payment is actually made. Therefore, there will be a time difference between the purchase and the payment in the record. However, we note that 97% of users in Money Forward ME link at least one credit card to the app, implying that usage of an unlinked credit card could be less frequently.

7 Money Forward ME automatically records all the transactions of banking accounts, including both inflows and outflows of money. Therefore, we also have information on incomes in addition to expenditures.

8 Figure 7 shows a comparison between the age distribution in our sample and that in census data.

9 In Money Forward ME, whether the household links only the first earner’s bank account or both the first and second earners’ is up to users, while the FIES tracks all the household members’ income information.

10 We cancel out bank transfers from one’s own bank account to another owned bank account if the two accounts are registered at Money Forward ME.

11 Note that this consumption hike in the week of SCP payment for Money Forward ME users may be sharper than for other households. Right after the SCP deposit, the system automatically sent notifications on the users’ smartphones. They were sent as general notifications about a large transaction. From July 15, Money Forward ME started to show special notifications. Municipalities also sent notifications mainly by mail, but the timing was not uniform across local offices.

12 In Broda and Parker (2014)’s household survey, the authors determine the liquidity constraint status by asking “In case of an unexpected decline in income or increase in expenses, do you have at least two months of income available in cash, bank accounts, or easily accessible funds?” In a similar survey by Coibion et al. (2020), the question is “Suppose that you had to make an unexpected payment equal to one month of your after-tax income, would you have sufficient financial resources (access to credit, savings, loans from relatives or friends, etc.) to pay for the entire amount?”

13 This result is possibly due to the strong cash demand of liquidity-constrained households for daily goods. We look at the difference in food and necessities consumption depending on liquidity constraints. However, the statistical results are unclear. This may be because those daily goods tend to be purchased by cash, which is not explicitly observed in the data.

14 The sum of the coefficients of durable goods is about 0.01 with 4 weeks after the SCP payment in Fig. 5d. Given that the average SCP payment is 232, 589 Japanese yen, durable good expenditure increased by 2300 yen. Since the SCP payment was concentrated in June and July, we expect that about 1000 yen was spent in each month, and given the average monthly durable good consumption, 6914 yen, we estimate the increase in durable goods as about 15%.

15 For a single-person household, the cutoff of monthly income for residential tax exemption was 100,000 Japanese yen. This cutoff increased by 50,000 Japanese yen with each additional household member.
